# “Is there a doctor on board?”: willingness and confidence of physicians in the Kingdom of Saudi Arabia in assisting with in-flight medical emergencies

**DOI:** 10.1186/s12873-021-00453-z

**Published:** 2021-04-30

**Authors:** Nouf A. AlShamlan, Reem S. AlOmar, Majd Mohammed Alrayes, Saud K. Alkhaldi, Ali Hamad Alomar, Abdulrahman Abdulaziz Alghamdi, Fares Mohammad Nassef, Sarah Hussain Al-Matar, Hatem A. Alqahtani

**Affiliations:** 1 Department of Family and Community Medicine, Imam Abdulrahman Bin Faisal University, Dammam, Saudi Arabia; 2 College of Medicine, Imam Abdulrahman Bin Faisal University, Dammam, Saudi Arabia; 3 College of Medicine, Dar Al Uloom University, Riyadh, Saudi Arabia

**Keywords:** In-flight, Medical, Emergency, Physician, Confidence, Willingness

## Abstract

**Background:**

In-flight medical emergencies (IMEs) are common, and for a traveling physician, it is very likely to encounter such a condition. Data discussing this issue are limited. Thus, this study aimed to evaluate the willingness and confidence of physicians in the Kingdom of Saudi Arabia (KSA) in responding to IMEs. As well as, to assess the associated sociodemographic, occupational, and travel-related factors, and their previous experience with such events.

**Methods:**

This cross-sectional, online-based, study was conducted among all physicians in KSA during January 2021. The self-administered questionnaire included questions on sociodemographic, occupational, travel profiles, willingness and confidence towards IMEs. Chi-Squared or Fisher’s Exact test were used for bivariate analysis followed by the multivariable binary logistic regression analysis.

**Results:**

A total of 4558 physicians participated in the study. About one-third of participants reported one or more IME incidents, and the vast majority of them provided assistance. Cardiovascular diseases were the most common IMEs. About half of the participating physicians are concerned about the medico-legal consequences of providing assistance with such a condition. Among all specialties, emergency physicians reported the highest willingness and confidence toward IMEs. Predictors for a physician’s willingness to assist in IMEs were being male, having been involved in a previous IME situation, attended life support and IME courses, frequent traveling, and practicing medicine in the Central region of Saudi Arabia.

**Conclusion:**

Findings from the current study stressed the need for establishing standardized guidelines about the roles of healthcare workers and the legal consequences of providing medical assessment in IMEs. Moreover, training programs on IMEs to all physicians, especially those who deal with a variety of cases during their practice such as internal medicine and family medicine are also suggested.

## Background

Around 2.75 billion passengers use commercial airlines annually worldwide. Safety concerns might arise when medical-related emergencies occur in the middle of the air, where access to medical assistance is limited. It is not uncommon for such incidents to occur, as it has been reported that there is one medical emergency for every 604 flights [1]. For a traveling physician, it is very likely to face such situations, where they might be called for medical assistance while they are on board. A retrospective review of records of calls for in-flight medical emergencies (IMEs) from five airlines to a doctor-directed consultations’ center between 2008 to 2010 reported that the most common medical problems encountered were syncope and pre-syncope (37.4%), followed by respiratory symptoms (12.1%), then nausea and vomiting (9.5%). Moreover, in 48.1% of the cases, a physician passenger provided assistance [1]. Another study found that between 1999 to 2000, there were 22.6 IMEs reported per million passengers, which accounted for about 210 diversions per million flights. The hospitalization rate was 49% in cases that were evaluated and decided to divert by a physician, in comparison to 15% in cases with no physician participation [2]. A cross-sectional study was conducted in Malaysia on 182 primary care physicians to assess their attitudes, knowledge, and confidence in dealing with in-flight emergencies. It was reported that only 11.5% of participants felt confident in dealing with IMEs. On the other hand, most participants (69.2%) would help if needed. However, the readiness to help was reduced if someone else already provided assistance or if they were not familiar with the case. Moreover, a higher knowledge score of IMEs was positively associated with higher confidence in managing these cases [3].

Globally, several reviews and case reports examining IMEs exist [4, 5]. However, there is a lack of comprehensive studies concerning the readiness and confidence of the traveling physicians to intervene in such in-flight emergencies, and to the best of our knowledge, none have been published in the Arab world, and in the Kingdom of Saudi Arabia (KSA) specifically. Thus, this study aimed to evaluate the willingness and confidence of physicians in KSA in responding to an IME. As well as, to assess the associated sociodemographic, occupational, and travel-related factors, and their previous experience with such events.

## Methods

The Institutional Review Board Committee of Imam Abdulrahman Bin Faisal University approved the study. Confidentiality and anonymity were assured. Written informed consent was obtained from participants**.** The study protocol is performed in accordance with the relevant guidelines.

### Study design

This cross-sectional study was performed among physicians with different specialties across KSA during January 2021.

### Study participants and sample size calculation

Physicians of both genders were included in the study. The minimum required sample size was calculated to be 1066. This is assuming a prevalence of 50% of physicians willing to help in an in-flight emergency. Given a precision of 3%, and at an alpha level of 0.05. Sample size calculation was done through Epi info 7.0. The minimum required sample was increased by 10% to overcome any possible potential missing values.

### Data collection tool and process

The data was collected by an online-based, self-administered questionnaire developed by the researchers after a review of recent literature with similar objectives of the current study. The survey has two main parts; the first part covers questions on sociodemographic, occupational, and travel profiles. The second part contains questions about willingness and confidence towards IMEs that were used by Katzer et al. and Ng WL et al. studies [3, 6]. This part had 11 items and responses to each item were reported on a 5-point Likert scale. A pilot study on 20 doctors excluded from the sample to ensure clarity of questions was used. After the pilot study, no major modifications in the questions were made. Two experts reviewed the questionnaire to enhance the content validity and all of them approved it. The online link of the survey was distributed to physicians through their registered emails in the Saudi Commission for Health Specialties (SCFHS). To avoid duplication of responses, the link did not accept multiple responses from the same participant.

### Statistical analysis

After checking for completeness and consistency, data were analyzed using IBM SPSS for Windows, version 26 (IBM Corp., Armonk, NY, USA). Categorical variables, presented as percentages and frequency distributions, were compared using the Chi-squared or Fisher’s exact tests. Figures were used to illustrate the responses of participants. The overall willingness and confidence scores were constructed by summation of the responses of the 5-point Likert-type scale, ranging from “strongly disagree” to “strongly agree”, after reverse coding of negatively framed statements. A high score was defined as a value at or above the 90th percentile. Multivariable binary logistic regression analysis was conducted to identify the independent predictors of the willingness to provide in-flight emergency care. Candidate variables were selected based on medical literature and bivariate analyses. Odds ratio (OR) with 95% confidence interval (CI) were estimated using the full model fit and were reported in comparison with the designated reference group. The presence of multicollinearity was detected through the bivariate Spearman’s correlation coefficients. The goodness-of-fit of the model was evaluated using the Omnibus and Hosmer-Lemeshow tests. The significance level was defined as α = 0.05.

## Results

### Characteristics of participants

The survey was completed by 4558 physicians, including 2557 (56.1%) men and 2001 (43.9%) women. Overall, 2490 (54.6%) participants were below the age of 30 years. Resident physicians and medical interns represented 30.5 and 27.3% of all participants, respectively. Over half (54.8%) of participants had a clinical experience of less than five years. The respondents included physicians from all regions of KSA. In total, 1976 (43.4%) of participants hold a Saudi board certification.

**Fig. 1:**
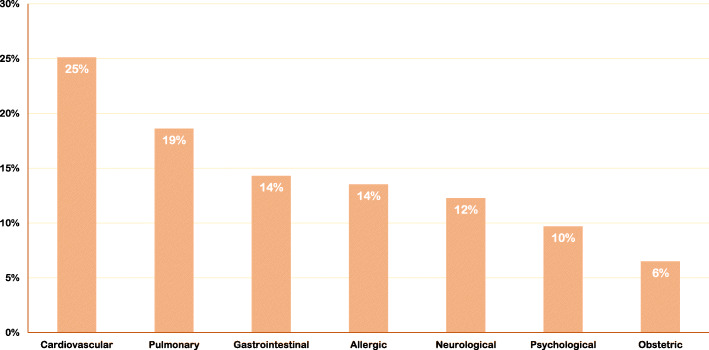
Types of inflight emergencies encountered by participants

As shown in Table 1, nearly three-fourths (75.6%) of participants reported having at least one flight per year, with 1467 (32.2%) participants reported having 2–3 flights per year. One-third (33.3%) of participants reported that they encountered at least one emergency during their previous flights. However, 321 (21.3%) of these participants did not provide their medical assistance in those emergencies. While only 915 (20.1%) participants attended an in-flight emergency course, the majority (88.7%) of participants attended life support courses, including Basic Life Support (86.0%) and Advanced Cardiac Life Support (45.4%) courses. Notably, participants who attended Advanced Life Support Courses had a higher tendency to provide medical assistance during in-flight emergencies than those who attended Basic Life Support courses only (83.1% vs. 74.9%) (*P* < 0.01). Figure 1 illustrates the types of in-flight emergency conditions encountered by participants. Cardiovascular (25.0%) and pulmonary (19.0%) conditions represented the most frequently encountered emergencies.

**Table 1:** Sociodemographic and travel characteristics of participants

Variable		Male		Female		Overall	
		N	(%)	N	(%)	N	(%)
Age (years)	< 30	1311	(51.3)	1179	(58.9)	2490	(54.6)
	30–39	663	(25.9)	495	(24.7)	1158	(25.4)
	40–49	283	(11.1)	176	(8.8)	459	(10.1)
	50–59	213	(8.3)	128	(6.4)	341	(7.5)
	≥ 60	87	(3.4)	23	(1.1)	110	(2.4)
Professional Rank	Medical Intern	611	(23.9)	661	31.6	1244	(27.3)
	Service Job	254	(9.9)	186	9.3	440	(9.7)
	Resident	793	(31.0)	596	29.8	1389	(30.5)
	Specialist	431	(16.9)	296	14.8	727	(15.9)
	Consultant	468	(18.3)	290	14.5	758	(16.6)
Region of Practice	Eastern Province	558	(21.8)	454	(22.7)	1012	(22.2)
	Central Province	859	(33.6)	628	(31.4)	1487	(32.6)
	Western Province	157	(6.1)	107	(5.3)	264	(5.8)
	Southern Province	735	(28.7)	646	(32.3)	1381	(30.3)
	Northern Province	248	(9.7)	166	(8.3)	414	(9.1)
Years of Experience	< 5	1344	(52.6)	1152	(57.6)	2496	(54.8)
	5–9	421	(16.5)	363	(18.1)	784	(17.2)
	10–15	273	(10.7)	205	(10.2)	478	(10.5)
	≥ 15	519	(20.3)	281	(14.0)	800	(17.6)
Board Certificate	Saudi Arabia	1116	(43.6)	860	(43.0)	1976	(43.4)
	Arab Country	171	(6.7)	104	(5.2)	275	(6.0)
	North America	123	(4.8)	62	(3.1)	185	(4.1)
	Europe	121	(4.7)	61	(3.0)	182	(4.0)
	Others	73	(2.9)	37	(1.8)	110	(2.4)
	Not Applicable	953	(37.3)	877	(43.8)	1830	(40.1)
Travel Frequency	< 1/year	589	(23.0)	522	(26.1)	1111	(24.4)
	1/year	566	(22.1)	500	(25.0)	1066	(23.4)
	2–3/year	842	(32.9)	625	(31.2)	1467	(32.2)
	> 3/year	450	(17.6)	270	(13.5)	720	(15.8)
	Monthly	110	(4.3)	84	(4.2)	194	(4.3)
Encountered Inflight Emergencies	Yes	866	(33.9)	650	(32.5)	1516	(33.3)
	No	1691	(66.1)	1351	(67.5)	3042	(66.7)
Attended Inflight Emergency Course	Yes	478	(18.7)	437	(21.8)	915	(20.1)
	No	2079	(81.3)	1564	(78.2)	3643	(79.9)
Attended Life Support Course	Yes	2255	(88.2)	1787	(89.3)	4042	(88.7)
	No	302	(11.8)	213	(10.7)	516	(11.3)
Basic Life Support	Yes	2194	(85.8)	1728	(86.4)	3922	(86.0)
	No	363	(14.2)	273	(13.6)	636	(14.0)
Advanced Cardiac Life Support	Yes	1292	(50.6)	775	(38.7)	2068	(45.4)
	No	1264	(49.4)	1226	(61.3)	2490	(54.6)

*Abbreviations: N:* Number of participants

### Willingness and confidence to provide medical Care in in-flight Emergencies

**Fig. 2:**
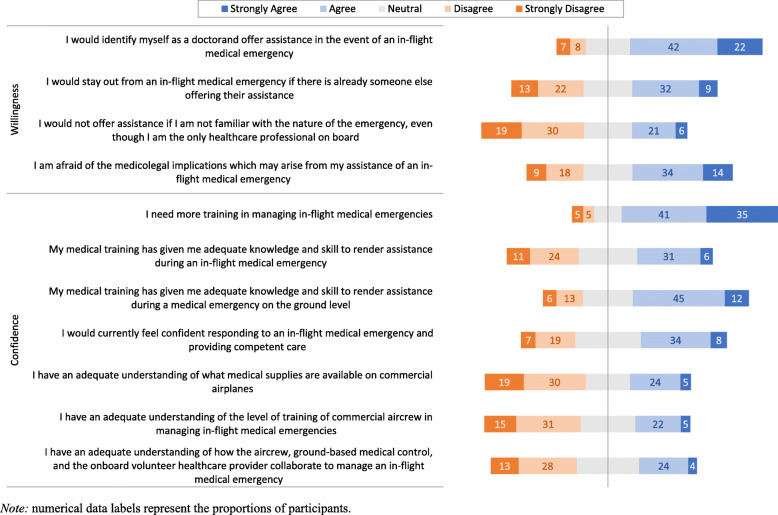
Participants responses on their willingness and confidence in inflight emergency medical assistance

Figure 2 summarizes the responses of participants on the 11 Likert-type statements on the willingness and confidence of participants to provide medical care during in-flight emergencies. Only 646 (15%) participants reported that they will not identify themselves as doctors in the event of an in-flight medical emergency. However, nearly half of the participants (2217, 48.7%) agreed or strongly agreed that they are afraid of the medicolegal implications which may arise from their assistance.

Regarding the confidence of participants in providing medical assistance in the event of IMEs, only 490 (10.8%) participants reported that they do not need more training in managing IMEs. While 2568 (56.3%) participants agreed or strongly agreed that their medical training has given them adequate knowledge and skills to provide assistance during emergencies on the ground level, 1907 (41.9%) participants reported having the adequate knowledge and skills to provide the assistance during in-flight emergencies.

**Fig. 3:**
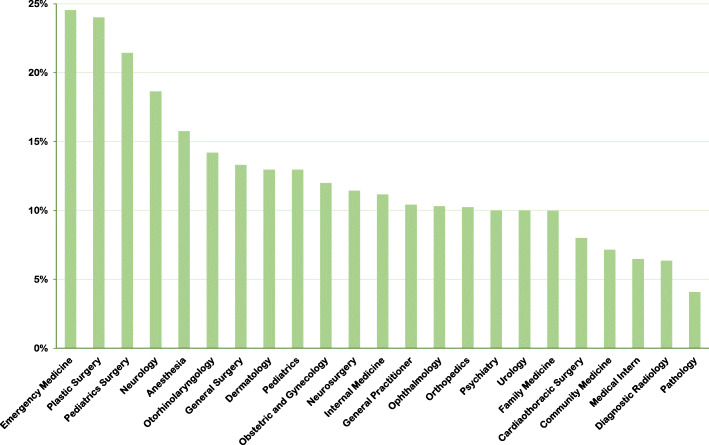
Participants with high willingness to provide infight emergency care according to specialty

**Fig. 4:**
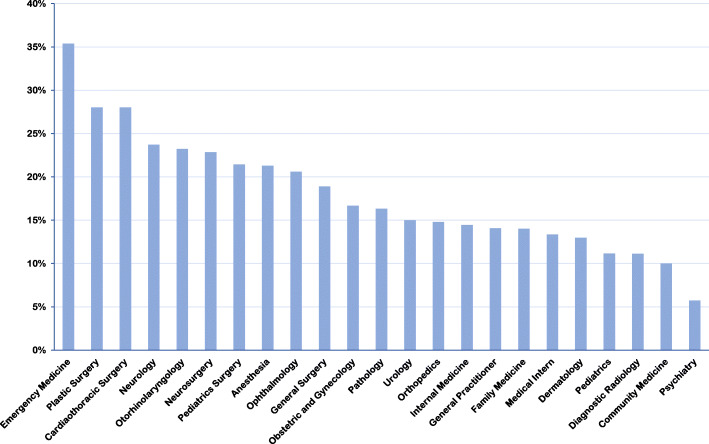
Participants with high confidence to provide infight emergency care according to specialty

Figures 3 and 4 illustrate the willingness and confidence of participants to provide medical assistance during IMEs according to their specialty. Unsurprisingly, emergency medicine physicians reported the highest willingness and confidence among all participants. In descending order, pathologists, diagnostic radiologists, and medical interns showed the lowest willingness to provide medical care. Additionally, psychiatrists, community medicine physicians, and diagnostic radiologists had the lowest confidence in IMEs.

### Factors associated with willingness and confidence in providing in-flight emergency care

Table 2 summarizes the association of willingness and confidence of physicians in providing in-flight emergency care according to different demographic and socioeconomic factors. Male physicians had a higher willingness (12.7% vs. 7.9%) and confidence (18.3% vs. 12.4%) in providing emergency care compared with female physicians (*P* < 0.01). The year of experience was found to be significantly associated with the willingness and confidence of participants (*P* < 0.05). For example, only 12.1% of physicians with less than five years of experience were in the high confidence group compared with 47.1% of physicians with more than 20 years of experience. Additionally, physicians practicing in the Central Province of KSA had the highest tendency to have a high willingness (12.7%) and confidence (19.3%) in providing care in in-flight emergencies compared with other physicians (*P* < 0.01). Furthermore, there was a significant association between the country of board certification and the willingness and confidence, where physicians who were board-certified in Europe and North America had the highest willingness (19.2%) and confidence (23.8%), respectively. Participants who encountered a previous in-flight emergency had a higher willingness (14.5% vs. 8.7%) and confidence (22.8% vs. 12.2%) in providing emergency care than their counterparts (*P* < 0.01). Similarly, participants who had attended the in-flight emergency course had higher confidence than those who did not (34.4% vs. 11.1%).

**Table 2:** Willingness and confidence of participants to provide care in in-flight emergencies according to sociodemographic and travel profiles

Variable		High Willingness		***P*** value	High Confidence		***P*** value
		N	(%)		N	(%)	
Age (years)	< 30	214	(8.6)	**< 0.01**	314	(12.6)	**< 0.01**
	30–39	128	(11.1)		191	(16.5)	
	40–49	57	(12.4)		109	(23.7)	
	50–59	60	(17.6)		84	(24.6)	
	≥ 60	25	(22.7)		19	(17.3)	
Gender	Male	326	(12.7)	**< 0.01**	468	(18.3)	**< 0.01**
	Female	158	(7.9)		249	(12.4)	
Professional Rank	Medical Intern	218	(8.7)	**< 0.01**	165	(13.3)	**< 0.01**
	Service Job	79	(10.1)		78	(17.7)	
	Resident	63	(13.2)		198	(14.3)	
	Specialist	33	(11.0)		137	(18.8)	
	Consultant	91	(18.2)		139	(18.3)	
Region of Practice	Eastern Province	98	(9.7)	**0.01**	110	(10.9)	**< 0.01**
	Central Province	189	(12.7)		287	(19.3)	
	Western Province	29	(11.0)		39	(14.8)	
	Southern Province	121	(8.8)		205	(14.8)	
	Northern Province	47	(11.4)		76	(18.4)	
Years of Experience	< 5	120	(7.4)	**< 0.01**	303	(12.1)	**< 0.01**
	5–9	188	(12.8)		130	(16.6)	
	10–15	105	(13.3)		103	(21.5)	
	≥ 15	191	(17.9)		181	(47.1)	
Board Certificate	Saudi Arabia	213	(10.8)	**< 0.01**	291	(14.7)	**< 0.01**
	Arab Country	52	(18.9)		48	(17.5)	
	North America	29	(15.7)		44	(23.8)	
	Europe	35	(19.2)		40	(22.0)	
	Others	15	(13.6)		37	(33.6)	
	Not Applicable	140	(7.7)		257	(14.0)	
Travel Frequency	< 1/year	69	(6.2)	**< 0.01**	194	(17.5)	**0.04**
	1/year	116	(10.9)		150	(14.1)	
	2–3/year	167	(11.4)		210	(14.3)	
	> 3/year	100	(13.9)		130	(18.1)	
	Monthly	32	(16.5)		33	(17.0)	
Encountered Inflight Emergencies	Yes	220	(14.5)	**< 0.01**	346	(22.8)	**< 0.01**
	No	264	(8.7)		371	(12.2)	
Attended Inflight Emergency Course	Yes	101	(11.0)	0.65	315	(34.4)	**< 0.01**
	No	383	(10.5)		402	(11.0)	
Attended Life Support Course	Yes	459	(11.4)	**< 0.01**	587	(14.5)	**< 0.01**
	No	25	(4.8)		130	(25.2)	
BLS Course	Yes	444	(11.3)	**< 0.01**	589	(15.0)	**< 0.01**
	No	40	(6.3)		128	(20.1)	
ACLS Course	Yes	296	(14.3)	**< 0.01**	423	(20.5)	**< 0.01**
	No	188	(7.6)		294	(11.8)	

*Abbreviations: N:* Number of participants; *BLS*: basic life support; *ACLS*: advanced cardiac lift support

### Multivariable analysis of factors associated with willingness to provide in-flight emergency care

Multivariable binary logistic regression analysis was performed to identify the factors associated with the willingness to provide medical assistance in the event of in-flight emergencies. The model revealed that participants who attended life support courses were 2.4-times more willing to provide medical assistance than those who did not (*OR* = 2.4; 95% *CI*: 1.6–3.7). Furthermore, male gender (*OR* = 1.6; 95% *CI*: 1.3–2.0) and having a previous encounter of in-flight emergency situation (*OR* = 1.5; 95% *CI*: 1.2–1.9) were found to be independent predictors of having a higher willingness to provide medical assistance. Participants who were board-certified in Europe and Arab Countries were 1.8-times more likely to provide medical assistance. Additionally, participants who had monthly (*OR* = 1.9; 95% *CI*: 1.2–3.0) or 2–3 flights/year (*OR* = 1.5; 95% CI: 1.1–2.0) had higher willingness to provide emergency care than those who traveled less than once a year (Table 3). Physicians practicing in the Central Province were 1.5-times more willing to provide medical assistance.

**Table 3:** Multivariable analysis of willingness of participants to provide care in in-flight emergencies

Variable		Willingness to Provide Infight Emergency Care		
		OR	[95% CI]	***P*** value
Male Gender		1.6	[1.3–2.0]	**< 0.01**
Province of Practice	Eastern	*Reference Group*		
	Central	1.5	[1.1–1.9]	**< 0.01**
	Western	1.2	[0.7–1.8]	0.49
	Southern	1.0	[0.7–1.3]	0.80
	Northern	1.3	[0.9–1.9]	0.21
Years of Experience	< 5	*Reference Group*		
	5–9	1.0	[0.7–1.3]	0.71
	10–15	1.1	[0.8–1.5]	0.63
	≥ 15	1.1	[0.8–1.5]	0.57
Board Certificate	Saudi Arabia	*Reference Group*		
	Arab Country	1.8	[1.2–2.6]	**< 0.01**
	North America	1.2	[0.8–2.0]	0.35
	Europe	1.8	[1.2–2.8]	**< 0.01**
Travel Frequency	< 1/year	*Reference Group*		
	1/year	1.6	[1.2–2.2]	**< 0.01**
	2–3/year	1.5	[1.1–2.0]	**0.01**
	> 3/year	1.7	[1.2–2.4]	**< 0.01**
	Monthly	1.9	[1.2–3.0]	**< 0.01**
Encountered Inflight Emergencies		1.5	[1.2–1.9]	**< 0.01**
Attended Life Support Course		2.4	[1.6–3.7]	**< 0.01**

*Abbreviations: CI*: confidence interval; *OR*: odds ratio

## Discussion

The findings from the current study revealed that about one-third of the sampled physicians in KSA encountered at least one incident of IME, and most of them provided medical assistance. Moreover, the vast majority of participants in our study reported that they will identify themselves as doctors in the event of IMEs. This finding agreed with a study among 182 primary health care physicians in Malaysia which reported that about 70% of participants were willing to help during IMEs [3].

Findings from this study revealed that cardiovascular (25.0%) and pulmonary (19.0%) conditions represented the most frequently encountered emergencies. Published data varied in reporting the incidence of emergency conditions that were encountered in flight, and cardiovascular conditions accounted for 40 to 14% of in-flight emergency cases [7, 8].

The current study revealed that only 10.8% of participants reported that they do not need more training in managing in-flight medical emergencies. Moreover, 56.3 and 41.9% of participants agreed that their medical training has given them adequate knowledge and skills to aid during emergencies at ground level, and during in-flight emergencies, respectively. This discrepancy between ground and in-flight emergencies is likely due to the inability to consult other physicians during IMEs in addition to the lack of facilities and equipment needed to diagnose and manage patients with such conditions [9].

Unsurprisingly, emergency medicine physicians in this study reported the highest willingness and confidence towards IMEs among all participants. This is most probably due to the nature of training and practice of emergency physicians which prepares them with the knowledge and skills needed to handle emergencies of any kind, whether they are on the ground or in flight.

Several challenging factors could affect a physician’s decision to participate in an IME and his/her confidence level, such as lack of expertise, uncertainty of diagnosis and management, the difficulty of managing patients in restricted surroundings, and doubt regarding the adequacy of care [4]. A factor found in this study was gender. In the current sample, male physicians had a higher willingness and confidence in providing IME care compared to female physicians. In a study that examined the behaviour of physicians with regards to providing medical emergency help outside their duties in North Carolina, gender was not considered as a factor [10]. Neither was it a factor amongst practicing internists in New York [11]. Considering that the sample from the current study comes from a sociocultural conservative population, such a factor may truly have played a role. Years of experience was also a significant factor in this study, where the more the experience the more the willingness and confidence of physicians to act on IMEs. In line with these findings, the current study has also identified that a previous encounter of an IME as well as attending an IME course and/or life support course were associated with the willingness and/or confidence of physicians. Similar results have been reported in the US [11]. Certainly, hesitation to declare oneself as a physician when asked on-board has been reported previously among young doctors [5]. However, lack of experience may not be the only reason behind hesitation in the current population. Fear of medico-legal ramifications may also have played a role [10]. This study showed that nearly half of the participants were concerned about the medico-legal implications which may arise from their assistance in IMEs. This was lower than reported by Ng et al. among 182 primary care doctors in Malaysia in which 62.6% of participants were afraid and 21.4% were unsure of the medico-legal consequences of their help in such a condition [3]. A physician who volunteers to provide medical assistance creates a doctor-patient relationship, and with it comes its obligations and liability risk. Generally, liability ensues from the country in which the aircraft is registered. However, the law of the country where the incident had occurred and in which the patient is a citizen may arguably be applied [12]. The ambiguity surrounding these issues could have added to such hesitation, and this finding stressed the need of establishing standardized guidelines about the roles of healthcare workers and the consequences of providing medical assessment in the IMEs.

Regional differences in physicians’ willingness and confidence to assist in IME situations were observed in the current study. Both in terms of board certifications abroad, and experience locally. Current results show that physicians who are board-certified from European and Arab countries were more likely to assist compared to those who are Saudi board certified. This may be explained by the frequent international travels to European and Arab countries for board training. Such frequent traveling was found to be a predictor in this study too. Frequent traveling may equip the prospective good Samaritan with knowledge about the aircraft’s whereabouts, hence more confidence in dealing with an emergency situation in a familiar setting. Local regional differences in willingness and confidence of physicians according to the region of clinical experience were also found in this study. Physicians practicing in the Central region of Saudi Arabia were more likely to assist in an IME situation. The Central region holds the capital of the country, Riyadh, which houses several medical cities and renown secondary and tertiary hospitals with plenty of medical education opportunities, which makes it unique to other regions of the country.

The current study – to our knowledge – is the first ever to examine physicians’ willingness and confidence to assist in IME situations. Indeed, the lack of literature on this topic – although seen as a strength – has also limited the ability to make comparisons with other studies. Few papers have discussed Good Samaritans, coming across IME very briefly. Furthermore, a considerably large sample size of physicians participated in this study allowing for advanced statistical analyses that have adjusted for possible confounders and which would make inferences to the entire physician population possible.

## Conclusion

This cross-sectional Saudi-based study has shown that the vast majority of participating physicians will identify themselves in an IME situation. It has also identified several predictors for a physician’s willingness and confidence to assist in such situations, namely, being male, having been involved in a previous IME situation, attended life support and IME courses, frequent traveling, and practicing medicine in the Central region of Saudi Arabia. About half of the participating physicians have reported fear of medico-legal ramifications surrounding such emergencies. Therefore, it is recommended that such issues be made clear by health officials. Moreover, providing training on IMEs to all physicians, especially those who deal with a variety of cases during their practice such as internal medicine and family medicine is also suggested.

## Data Availability

The datasets used and/or analyzed during the current study are available from the corresponding author on reasonable request.
